# A Zero-Shot Learning Approach for Blockage Detection and Identification Based on the Stacking Ensemble Model

**DOI:** 10.3390/s24175596

**Published:** 2024-08-29

**Authors:** Chaoqun Li, Zao Feng, Mingkai Jiang, Zhenglang Wang

**Affiliations:** 1 Faculty of Information and Automation, Kunming University of Science and Technology, Kunming 650500, China; 20222104026@stu.kust.edu.cn (C.L.); 20222104025@stu.kust.edu.cn (Z.W.); 2 Yunnan Key Laboratory of Intelligent Control and Application, Kunming University of Science and Technology, Kunming 650500, China; 3 Guangzhou Nansha Power Supply Bureau, Guangdong Power Grid Limited Liability Company, Guangzhou 511458, China; jiangmingkai1@hotmail.com

**Keywords:** zero-shot learning, defect detection, signal processing, ensemble learning, attribute description

## Abstract

A data-driven approach to defect identification requires many labeled samples for model training. Yet new defects tend to appear during data acquisition cycles, which can lead to a lack of labeled samples of these new defects. Aiming at solving this problem, we proposed a zero-shot pipeline blockage detection and identification method based on stacking ensemble learning. The experimental signals were first decomposed using variational modal decomposition (VMD), and then, the information entropy was calculated for each intrinsic modal function (IMF) component to construct the feature sets. Second, the attribute matrix was established according to the attribute descriptions of the defect categories, and the stacking ensemble attribute learner was used for the attribute learning of defect features. Finally, defect identification was accomplished by comparing the similarity within the attribute matrices. The experimental results show that target defects can be identified even without targeted training samples. The model showed better classification performance on the six sets of experimental data, and the average recognition accuracy of the model for unknown defect categories reached 72.5%.

## 1. Introduction

Due to rapid urbanization, underground pipe networks in urban areas have become increasingly large and complex, and as a result, drainage pipes have become a crucial part of urban infrastructure. As pipelines age and the environment changes, structural and functional failures such as leaks, blockages, and cracks often occur in drainage pipes, which can result in reduced water flow, negatively impacting the daily lives of residents, posing a risk to road safety, and potentially causing casualties [1]. Therefore, researching the identification of defects in drainage pipes and monitoring their operational status is of great significance, thus helping to prolong the service life of pipes, reduce economic losses, and eliminate potential safety hazards in urban areas.

Currently, the research on pipeline failures is mainly focused on leak detection. Leaks, as a common pipeline defect, can only be detected after the defect has already developed, and defects are identified by detecting changes in pressure and flow in the pipeline. Pipeline blockage is a gradual process; the flow rate in the pipeline at the beginning of the blockage will not appear to have changed much, and the pipeline can still operate normally, so leak detection methods do not apply to pipeline blockage detection. Data-driven defect detection methods have therefore received increasing attention in recent years, but they usually require many labeled samples, which is typically difficult to obtain in practical applications. Miao et al. [2] employed a semi-supervised learning method (a combination of metric learning and a pseudo-labeling strategy) to enhance the representation of features, and their experimental results showed that the method can effectively solve the current problem of limited supervised learning fault samples. Zuo et al. [3] proposed a semi-supervised pipeline fault diagnosis method by modifying the loss function of the long- and short-term memory autoencoder to reduce the dependence on data, achieving 98% accuracy on the experimental dataset in the process. Deng et al. [4] solved the problem of fault diagnosis under extremely limited label conditions by capturing fault features with an adversarial decoupled autoencoder (ADAE) with feature regularization, and then implementing fault diagnosis with a low-dimensional feature distance metric (LFDM). Wang et al. [5] combined the advantages of multiple models by inputting processed low-dimensional features into an optimized support vector machine model, achieving 100% classification accuracy for faults. Li et al. [6] combined a semi-supervised probabilistic support matrix machine (SPSMM) with infrared imaging to eliminate the problem of insufficient sample labeling. All of the abovementioned studies were conducted based on fault types with a certain number of labels. However, in engineering practice, due to the complexity and diversity of the external environment of pipeline operation, the labeled samples of certain fault types are difficult to obtain, and for these unlabeled data, there are no available data in the training set to train them. Thus, the traditional supervised learning method was invalidated. To solve these problems, the idea of zero-shot learning (ZSL) is introduced in the field of pipeline defect identification.

Zero-shot learning is derived from transfer learning and is a special case of transfer learning [7]. The major difference between zero-shot learning and transfer learning is that in zero-shot learning, the labels of the samples in the test set do not appear in the training set. Zero-shot learning was first proposed by Lampert and Palatucci et al. in 2009. A direct attribute migration method was proposed by Lampert [8]. In the same year, the concept of zero-shot learning was formalized by Palatucci et al. [9]. Zhang et al. [10] proposed a distribution- and gradient-constrained embedding model (DGEM) for solving the bias problem and overfitting problem in the zero-shot learning process and verified the effectiveness of DGEM on five ZSL datasets. Gull et al. [11] proposed a generative model based on identifiable VAE (iVAE) to solve traditional and generalized ZSL problems. In the above zero-shot learning methods, the experimental objects are mostly two-dimensional image data of human portraits and animals. However, the visual attributes of pictures are not the same as the one-dimensional time-domain data acquired by industrial sensors, and the use of the zero-shot learning method described above requires the image conversion processing of the one-dimensional time series signals. Therefore, in recent years, some scholars have begun to introduce zero-shot learning methods into the field of defect identification.

Industrial zero-shot learning models are mainly categorized as generative and embedded. Generative models convert a zero-shot learning problem into a supervised learning problem by learning the relationship between semantic descriptions and real feature distributions and using semantic descriptions of unknown classes to generate data or features for unknown classes. Xu et al. [12] used adversarial training between single-fault samples and LIV to generate load fault features and identified composite faults by measuring the distance between features extracted from test composite fault samples and features generated from LIV. Zhuo et al. [13] used an adversarial network to generate samples and introduced auxiliary loss in the form of a triad to jointly train the loss term. Lv et al. [14] solved the zero-shot classification problem with a mixed-attribute conditional adversarial denoising autoencoder (CADAE) conditioned on mixed attributes and verified the excellence of the method on three datasets. However, the quality of samples generated by generative models may be inconsistent, and generative models may tend to generate new samples that are similar to existing samples, resulting in a lack of diversity in the generated samples, which can limit the model’s ability to generalize to unknown classes.

The embedding method has to learn an embedding function, add auxiliary information for the unknown class of defects, establish attribute links between known and unknown samples, and finally achieve defect classification through a similarity measure. Feng et al. [15] first used zero-shot learning in the field of industrial fault diagnosis to solve the zero-shot fault diagnosis problem with artificially defined attribute descriptions. Sun et al. [16] used a domain adaptation measure to mitigate the projection domain bias problem, then adopted a scaling calibration strategy to avoid identification preferences for known defects, and finally demonstrated the reliability of the method on two datasets. Fan et al. [17] used the attribute fusion transmission (AFT) approach for zero-shot fault diagnosis and proved the reasonableness of the approach through comparison experiments and ablation experiments on publicly available datasets. Xu et al. [18] used convolutional neural networks to extract fault features, embed the fault features in the visual space, and finally used Euclidean distance to perform the metrics and achieve fault classification. Lin et al. [19] proposed a compatibility method based on nonlinear mapping (NMC), which can effectively classify samples of unknown classes. Xu et al. [20] constructed a low-dimensional semantic and convolutional autoencoder from collected vibration signals and defined an adaptive edge-centered loss in feature extraction to achieve the diagnosis of unknown composite faults.

The development of pipeline blockages is a gradual process, and new defect types are often derived from engineering practice. Traditional supervised learning methods are unable to categorize unknown classes. Therefore, this paper proposes a pipeline defect identification model based on stacking ensemble learning to address the above problems. A method of identifying unknown defect types in industrial scenarios is studied through attribute learning. Multiple attribute descriptions are provided for each defect type to establish attribute links between known and unknown categories. VMD is applied to decompose and denoise the original acoustic signals recorded by the sensor, and effective features are derived from the IMF components to construct the feature vectors. Second, six fully presented attributes are assigned to each pipeline operating state studied in this paper. The attribute matrix is then constructed from the corresponding attributes. Finally, an attribute learning model is built using the stacking ensemble structure. The base classifiers of the stacking model consist of multiple single machine learning models. The criterion for selecting a suitable base classifier is the high classification accuracy of the base classifiers and the distinct differences between them. Next, to avoid overfitting of the data, 10-fold cross-validation is applied to each of the selected base classifiers separately, and the results of the base classifiers are input into the meta-classification for further processing. The unknown defect identification is completed by calculating the similarity with all the class attribute labels. The main contributions to this work are as follows:

1. This paper proposes a zero-shot learning method for pipe defect identification based on the stacking ensemble and, for the first time, introduces the ensemble learning approach into the field of zero-shot defect identification. The proposed ensemble learning model takes into full consideration the differences between base classifiers and the accuracy of each one. The adoption of diverse base classifiers can enhance the ensemble model’s diversity and overcome the limitations of individual classifiers. The method enables the identification of unknown defect categories with only a small number of training samples. Furthermore, the proposed method is more accurate and robust compared to single machine learning models.
2. This paper demonstrates the feasibility of the proposed method by applying it to our experimental data. The method can identify pipeline defects (blockages at this stage of our research), while the sample labels included in the training set are completely different from those in the testing set. The issue of inaccessible sample labels in complex industrial scenarios, resulting in the inability to classify new defects, is resolved. The accurate categorization of pipeline blockage conditions is conducive to the complete assessment of pipeline operation status and failure risk, the timely adoption of necessary measures to avoid further expansion of hazards, and reductions in resource waste and equipment maintenance costs.

The rest of this paper is organized as follows: Section 2 introduces the related work of this paper; Section 3 introduces the theories related to the method proposed; Section 4 describes the source of the experimental data; Section 5 provides the specific process of the zero-shot defect identification method proposed and a series of comparative experiments on the existing dataset; and Section 6 concludes this paper.

## 2. Related Work

Traditional supervised learning requires many labeled pieces of data to be obtained, but this often requires significant human and financial resources. In engineering practice, the environment and conditions of equipment operation may be so complex that it is difficult to collect samples for certain defect types, or even new defect types may appear. For these defect types without labeled samples, zero-shot learning methods are proposed.

In zero-shot learning, the model will directly affect the final recognition. At present, the application scenarios of zero-shot learning are mainly divided into two categories: traditional zero-shot learning for image datasets and zero-shot learning for industrial scenarios. Traditional zero-shot learning methods establish attributes by extracting descriptions of images and visual features. Ye et al. [21] proposed an asymptotic ensemble network model to solve the zero-shot image recognition problem, and the effectiveness of the proposed model was demonstrated on several ZSL datasets. However, the time series data collected by industrial sensors do not have the visual properties of images, so this method is not effective for industrial data recognition. Zero-shot learning methods in industrial scenarios are mainly composed of deep learning and machine learning models. The deep learning-based method first needs to convert the one-dimensional time series data into a two-dimensional image and then use the deep network model for recognition. Zhang et al. [22] used a one-dimensional convolutional neural network to extract signal features, then constructed a semantic description, and finally used a bilinear compatibility function to achieve classification and identification of defects. Although deep learning models have become extremely popular in recent years, they require many data to support them and are not applicable to the problem of identifying buried pipeline defects in the small sample studied in this paper. Based on machine learning methods, the attribute migration of fault descriptions is used to solve the zero-sample fault diagnosis task, which can avoid the processing of data conversion. Feng et al. [15] used human-defined fault descriptions to determine fault categories and demonstrated the feasibility of this method in the Tennessee Eastman process and the real thermal power plant process. A single machine learning model facing a complex task, such as zero-shot defect identification, may have limited feature representation ability due to the complex correlation properties between features, which may reduce the diagnostic accuracy and robustness of the model.

Therefore, it is crucial to explore a zero-shot learning model that is more suitable for the specific scenario of underground drain defect identification.

## 3. Main Methods and Theories

### 3.1. Problem Formulation

Zero-shot learning is a method derived from transfer learning, but it differs from transfer learning in that the sample labels from the test set do not appear in the training set in zero-shot learning. The categories of the samples in the training set are called known categories and are denoted as $S=\left\{c_{i}^{s}|i=1,2,\ldots ,N_{S}\right\}$, where $N_{s}$ is the number of training categories. The training set can be denoted as $D^{tr}=\left\{x^{tr}\in \chi_{s},y^{tr}\in \gamma_{s}\right\}$. Each sample $x^{tr}$ corresponds to a label $y^{tr}$. For the zero-shot learning task, the training set is all from the known category S, and the test set is from the unknown category $U=\left\{c_{i}^{u}|i=1,2,\ldots ,N_{u}\right\}$, where $N_{u}$ is the number of test categories, and the test set can be represented as $D^{te}=\left\{x^{te}\in \chi_{u},y^{te}\in \gamma_{u}\right\}$ with no intersection of known and unknown categories. Each sample $x^{te}$ corresponds to a label $y^{te}$. The known and unknown categories have no intersection and can be represented as S∩U=∅. In addition, we provide six attributes for each defect category. Each fine-grained attribute can be used for both known and unknown defects.

**Figure 1:**
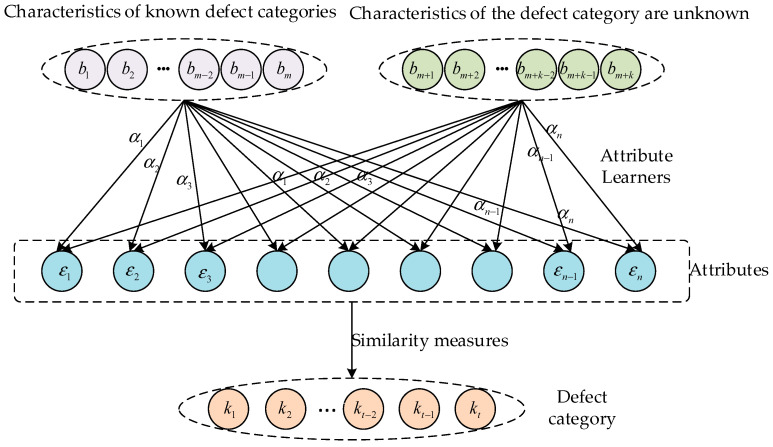
Zero-shot troubleshooting schematic.

As shown in Figure 1, $b_{1},b_{2},\cdot \cdot \cdot ,b_{m}$ are the extracted valid features for the known defect category, $b_{m+1},b_{m+2},\cdot \cdot \cdot ,b_{m+k}$ are the valid features extracted for the unknown defect category, and $\varepsilon_{1},\varepsilon_{2},\ldots ,\varepsilon_{n}$ is the information shared by the known and unknown defect categories, which we call attributes. In the module training phase, the attribute learner is trained with the known defect categories $\alpha_{1},\alpha_{2},\ldots ,\alpha_{n}$. In the testing phase, the trained attribute learner is used to predict the attributes of unknown defect categories. The classification process is as follows:

(1) $$f(x)=\underset{j=1,\cdot \cdot \cdot ,k}{\arg\max}\prod_{n=1}^{N}\frac{P\left(\varepsilon_{n}^{j}|x\right)}{P(\varepsilon_{n}^{j})},$$

where $P(\varepsilon_{n}^{j})$ is the prior estimate of the specific attribute of this class, $P\left(\varepsilon_{n}^{j}|x\right)$ is the probability of a specific attribute being included in the input data x, and k is the number of attributes.

The goal of zero-shot learning is to use the training set $D^{tr}$ to learn the mapping function f(x):χ→μ that can be applied to the test set, minimizing the error ζ, as shown below.

(2) $$f^{*}=\arg\min E_{(x,y)\in D^{te}}\zeta (f(x),y),$$

### 3.2. Model Structure

**Figure 2:**
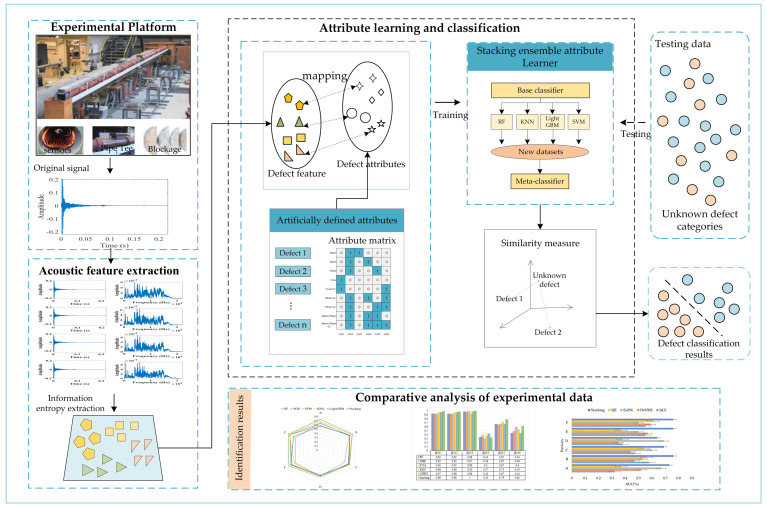
Framework of the proposed method.

Figure 2 illustrates the zero−shot pipeline defect identification based on the stacking ensemble proposed in this study. The technical route of this research consists of three parts: acoustic feature extraction, attribute learning and classification, and comparative analysis of experimental data. First, in the acoustic feature extraction stage, VMD was applied to decompose and denoise the original acoustic signals recorded by the sensor, and effective features were derived from the IMF components to construct the feature vectors. Secondly, in the attribute learning and classification stage, an attribute learning model was built using the stacking ensemble structure. In the module training phase, feature vectors extracted from known defect categories are used to train the attribute learner. In the testing phase, the trained attribute learner is used to predict unknown defect categories. Finally, a comparative analysis is performed on different datasets and using different methods. The following sections outline the steps required for each phase.

#### 3.2.1. Acoustic Feature Extraction

First, the acoustic signals collected from the experimental platform are analyzed in the time−frequency domain. Second, signals are decomposed using VMD. The number of decomposition layers is determined by the center frequency. Finally, the information of the signals in each layer is extracted via information entropy based on the IMF components to construct the feature vectors. Among them, the center frequency is the frequency point with the most concentrated energy in the frequency range and is a key parameter to describe the spectral characteristics of the signal. IMF is the component signal obtained after the original signal is decomposed, and each IMF represents a certain frequency component of the signal.

- Principle of VMD algorithm

**Figure 3:**
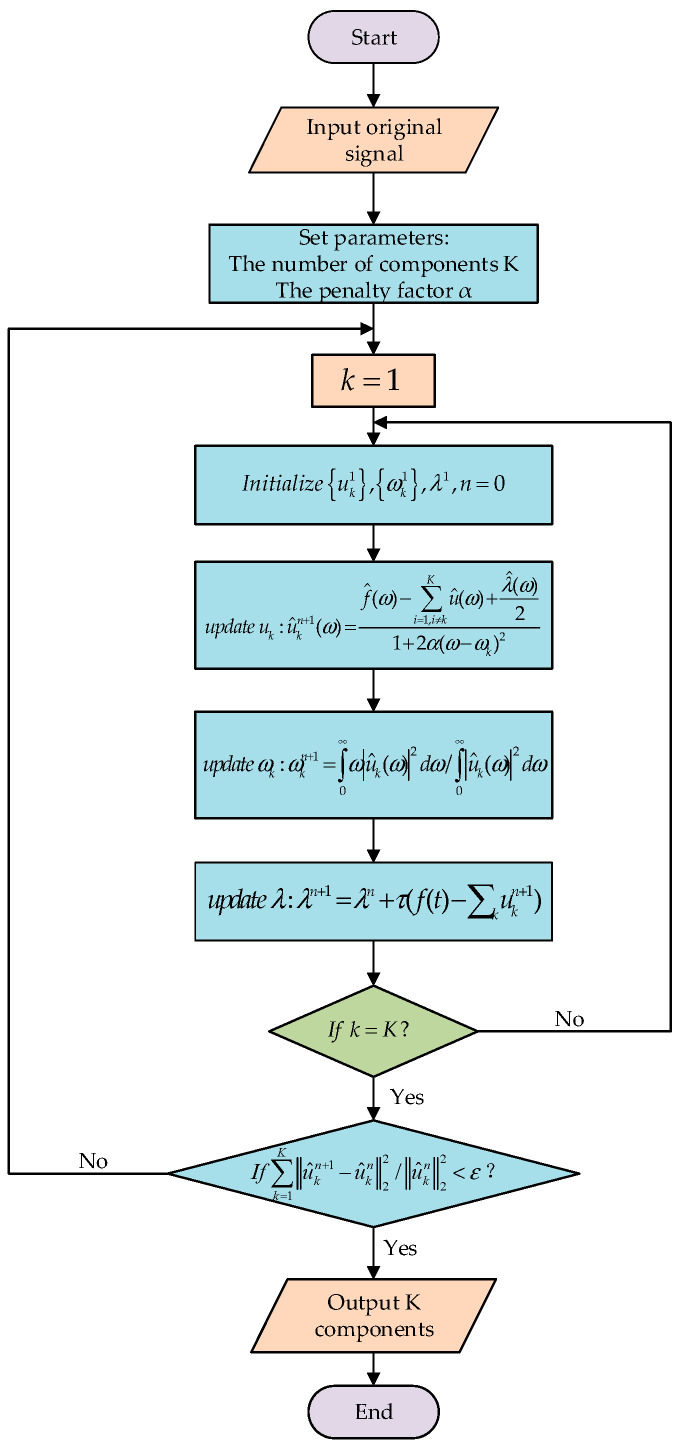
Flowchart of VMD algorithm.

VMD, proposed by Dragomiretskiy et al. in 2013, is an adaptive and non−recursive method that decomposes a signal into a finite number of IMF components. This method is especially suitable for analyzing and processing nonlinear, nonsmooth signals because it can not only adaptively determine the correlation band of each mode but also estimate the corresponding modes, which have been widely used in the processing of pipeline signals. The process of implementing the VMD algorithm is shown in Figure 3.

The main decomposition steps of VMD are as follows.

1. For each modal function $u_{k}(t)$, the Hilbert transform is used to compute the corresponding analytic signal.
2. Mixing $u_{k}(t)$ with the exponential term of the predicted center frequency modulates the spectrum of each mode into the corresponding fundamental frequency band.
3. The variational expression for solving the constraints is
  (3) $$\left\{\begin{array}{c} \min\left\{\sum_{k}{\left\|\partial_{t}\left[\left(\delta (t\right)+\frac{j}{\pi t})\times u_{k}(t)\right]e^{-j\omega_{k}t}\right\|}_{2}^{2}\right\}\\ s.t\sum_{k}u_{k}=f \end{array}\right.$$
4. Introducing the quadratic penalty factor α and the Lagrange multiplier operator λ(t) to find the optimal solution of the above equation changes the constrained variational problem into an unconstrained variational problem:
  (4) $$\begin{array}{l} L(\left\{u_{k}\right\},\left\{\omega_{k}\right\},\lambda ):=\alpha \sum_{k}{\left\|\partial_{t}\left[\left(\delta (t\right)+\frac{j}{\pi t})\times u_{k}(t)\right]e^{-j\omega_{k}t}\right\|}_{2}^{2}\\ +{\left\|f(x)-\sum_{k}u_{k}(t)\right\|}_{2}^{2}+\langle \lambda (t),f(t)-\sum_{k}u_{k}(t)\rangle \end{array}$$
  where $u_{k}$ represents the kth mode, $\omega_{k}$ represents the center frequency of the kth mode, and λ A is the Lagrange multiplier.
5. The Alternating Direction Method of Multipliers (ADMMs) algorithm is used to update $u_{k}$ and $\omega_{k}$:
  (5) $${\hat{u}}_{k}^{n+1}(\omega )=\frac{\hat{f}(\omega )-\sum_{i=1,i\neq k}^{K}\hat{u}(\omega )+\frac{\hat{\lambda }(\omega )}{2}}{1+2\alpha {\left(\omega -\omega_{k}\right)}^{2}},$$
  (6) $$\omega_{k}^{n+1}=\frac{\int_{0}^{\infty }\omega |{\hat{u}}_{k}(\omega ){|}^{2}d\omega }{\int_{0}^{\infty }|{\hat{u}}_{k}(\omega ){|}^{2}d\omega }$$
  where ${\hat{u}}_{k}^{n}(\omega ),\;\hat{f}(\omega ),\;\hat{\lambda }(\omega )$ are the Fourier transforms of $u_{k}(\omega ),f(\omega ),\lambda (\omega )$, respectively. n is the number of iterations.
6. After completing the update of K eigenmode functions, λ(ω) is updated:
  (7) $$\lambda^{n+1}=\lambda^{n}+\tau (f(t)-\sum_{k}u_{k}^{n+1})$$
  where τ denotes the noise tolerance parameter.

The iteration stops when $\sum_{k=1}^{K}{\left\|{\hat{u}}_{k}^{n+1}-{\hat{u}}_{k}^{n}\right\|}_{2}^{2}/{\left\|{\hat{u}}_{k}^{n}\right\|}_{2}^{2}<\varepsilon$.

- Information entropy

Entropy is a measure of the expected value of the occurrence of a random variable, while information entropy is a measure of how chaotic the distribution of a collection of samples is. The principle of information entropy is based on the probability distribution of information, which is calculated using the formula:

(8) $$H(X)=-\sum_{i=1}^{n}P(X=x_{i})\;\times \;\log_{2}P(X=x_{i})$$

where n is the number of possible values of the random variable, X denotes a random variable, and $P=(X=x_{i})$ is the probability that the random variable X takes $x_{i}$.

#### 3.2.2. Attribute Learning and Classification

In the experiment, seven out of the nine defect types are randomly selected for training, and the remaining two are used for testing. The dataset is separated into six parts. The divided training set serves as input training material for the stacking ensemble attribute learner. During the testing phase, the test set inputs for the trained attribute learner to obtain the attribute prediction matrix. The final classification is determined by measuring the degree of similarity with the attribute matrix of the defect type. This process identifies previously unknown class defect samples.

- Model Training Strategy

Stacking ensemble learning [23] is a type of heterogeneity that was first proposed by Wolpert. The learner consists of two layers of classifiers: the first being the base classifier and the second being the meta−classification. The data are first trained in the first layer of base classifiers, which processes the diagnostic data and obtains preliminary classification results. Then, the classification results of multiple base classifiers are used as inputs to meta-classifiers, and the classification results of meta-classifiers are used as the final prediction results [24]. However, if the meta-learner in the second layer directly uses the training results of the base learner in the first layer, overfitting will occur, leading to data reuse. To avoid overfitting the model during training, K-fold cross-validation is used. Although there is no rule for the choice of K value, 10 is usually used for small datasets, and 5 and 3 for medium and large datasets [25], respectively. Due to the limited data collected on the experimental platform, 10-fold cross-validation is used in this research.

For K-fold cross-validation, the defect sample set Y is first divided into K equally sized subsets of samples $Y_{1},Y_{2},Y_{3},\ldots Y_{K}$, and these K subsets are traversed in turn. Each time, the current subset is used as the verification set, and all the remaining samples are used as the training set to train and evaluate the model. Finally, the average of the K evaluation metrics is used as the final evaluation metric. Thus, every base classifier in the defect sample set Y is given a predicted value for a category. The classification results obtained from each base classifier are then combined to form a new sample set $Y_{\mathrm{new}}$. $Y_{\mathrm{new}}$ serves as the input to the meta-classifier, which ensembles the prediction results of the base classifiers again to obtain the final classification results.

- Selection of Base and Meta Classifiers

When using stacking ensemble learning, the selection of base classifiers will influence the results of ensemble model classification. Ensemble learning requires that each base classifier not only has a certain level of accuracy but also that there is a difference between each [26]. This is because a base classifier model with a higher level of learning accuracy improves the overall classification accuracy of the model, whereas the different types of base classifiers can combine the advantages of each classifier to overcome the limitations of a single model and improve the reliability of the overall model. Therefore, from the principle of the model, the method based on the probability statistics principle uses the naive Bayes (NB) model, the method based on the clustering principle uses the K-nearest neighbors (KNN) model, the method based on the kernel function principle uses the support vector machine (SVM) model, and the random forest (RF), light gradient boosting machine (LightGBM), and decision tree (DT) models are selected for the method based on the tree classifier principle.

For the selection of base classifiers, NB utilizes prior probability and is more effective in classifying small sample data. KNN [27] has better performance on nonlinear data since it outlines similarity between samples through K-nearest samples. SVM [28] focuses on support vectors in the vicinity of the decision boundary, showing better noise immunity and performing better on small sample datasets. RF [29] predicts through multiple decision trees and therefore has better noise immunity and classification accuracy. LightGBM [30] is more efficient in terms of training speed and does not occupy too many system resources. It can also reduce the residuals of the model through continuous iteration with good classification results. DT captures complex nonlinear relationships between features. In summary, this research selects NB, KNN, SVM, RF, LightGBM, and DT as base classifiers.

A suitable meta-classifier should have a simple structure and strong generalization ability. The extreme gradient boosting tree [31] has regular terms in the objective function. The model not only prevents data overfitting but also has high classification accuracy. XGBoost was therefore chosen as the meta-classifier for stacking ensemble learning.

#### 3.2.3. Comparative Analysis of Experimental Data

After completing the zero-shot defect identification task, we compared the proposed stacking ensemble approach with several machine learning models on six randomly divided datasets. The results prove the feasibility and reliability of the ensemble approach for zero-shot defect identification. To demonstrate the applicability of the method to one-dimensional time series data collected by industrial sensors, this study chose four traditional zero-shot learning models to perform the comparison.

## 4. Introduction to the Dataset

The data used in this research were obtained from the University of Bradford [32], UK. To simulate the functioning of real-world buried drainage pipes, a laboratory experiment was conducted involving the construction of a 15.4-m-long pipe with a diameter of 150 mm. The pipe was fabricated from concrete, and blockages within the pipe were artificially created using stones of 20 mm, 40 mm, and 55 mm in diameter, respectively.

The experimental setup comprised a microphone, speaker, filter, amplifier, sound card, and computer terminal. The speaker and microphone were positioned at the upstream end of the pipe, ensuring that both the speaker and microphone were aligned at the same height. The microphone was connected to the computer terminal via a filter, while the speaker was linked to the computer terminal through an amplifier and sound card. Blockages were strategically placed at the base of the pipe, with a baffle installed at the downstream end to concentrate sound energy. Windmills software was employed on a computer to control the sound card, generating a 10-s sinusoidal sweep signal ranging from 100 to 6000 Hz. The output from the sound card was amplified by a power amplifier and transmitted into the pipe via the speaker. As the sound waves propagated through the pipe, they encountered obstacles, resulting in the reflection and transmission of the waves. The reflected signals were captured by the microphone located at the upstream end of the pipe. Subsequent to this, the acquired waveback signals were filtered and transmitted to the computer for further processing and analysis. The filter employed had a frequency range of 100 to 4000 Hz, and the sampling rate was 44,100 Hz.

The acoustic measurement system employed in this study comprises four miniature microphones and a speaker. One of the four miniature microphones was mounted horizontally on a PCB circuit board. The speaker model K64WP5OHM is from the German Visaton company, and the microphone model SPM0208HE5 is from the American Knowles Acoustics company.. To ensure the precision of sound intensity measurements, the distance between the microphones must exceed the wavelength of the sound. The microphones were arranged with non-equal spacing to maximize the number of microphone pairs with unique inter-microphone distances, thus facilitating a broader frequency range for sound intensity measurements.

## 5. Experimental Results and Analysis

### 5.1. Signal Pre-Processing

**Figure 4:**
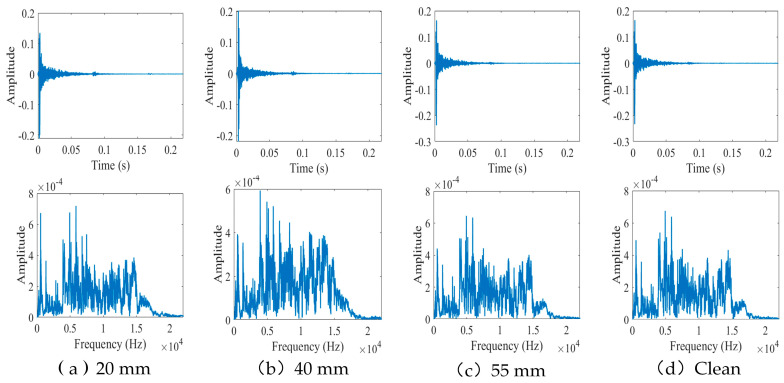
Time−frequency domain signal for the first four operating states of the pipeline. (**a**) 20 mm. (**b**) 40 mm. (**c**) 55 mm. (**d**) Clean.

The following nine pipeline operating states were experimented with and designed: normal empty pipeline, normal empty pipeline with a tee, pipeline with 20 mm blockage, pipeline with 40 mm blockage, pipeline with 55 mm blockage, pipeline with a tee and 40 mm blockage, pipeline with a tee and 55 mm blockage, pipeline with a tee and with 40 mm and 55 mm blockage, and pipes with both 40 mm and 55 mm blockages. In total, 50 datasets were collected for each pipe in each operating condition, totaling 450 (50 × 9) datasets. At the same time, this paper specifies that the ratio of the blockage and the inner diameter of the pipe is less than 1/5 for mild blockage, more than 1/3 for severe blockage, and between 1/5 and 1/3 for moderate blockage, hence the paper of 20 mm for mild blockage, 40 mm for moderate blockage, and 55 mm for severe blockage. The specific details are shown in Table 1:

The time and frequency domain diagrams of the acoustic signal cover the first four states of pipeline operation. The time−frequency domain diagram of the acoustic signal is shown in Figure 4:

As shown in Figure 4, the time−frequency plots of the acoustic signals are very similar because sound waves are reflected, diffracted, and transmitted between the pipe walls and blockages as they travel through the pipe. Moreover, the external environment inevitably affects the signal propagation in the medium. As a result, the signal collected by the sensor contains a significant amount of noise that obscures the characteristics of the acoustic signal and makes defect identification more challenging. To improve the accuracy of pipeline defect feature extraction, a VMD denoising algorithm was adopted to preprocess the acquired signals [33].

The steps for pre-processing acoustic signals are as follows. First, the initialization parameter (k) of the scale is set to 2, and the penalty parameter α is set to 2000 based on previous studies. Second, the VMD of the acquired signal is performed to obtain each component of the signal ($IMF_{1},IMF_{2},\ldots ,IMF_{K}$). Finally, this study also checked whether the center frequencies of the IMF component signals are similar. If so, the signal is considered to be over−decomposed [34], and the number of decomposition layers required is k. The center frequencies of each IMF component obtained are shown in Table 2 and Table 3 (in the case of a normal empty pipeline and a pipeline containing a 20 mm blockage). The tables indicate that the IMF value of the last component is the same when k equals 4 and 5. Since k=5 is considered to be an over−decomposition, the final number of decomposition layers is 4.

**Figure 5:**
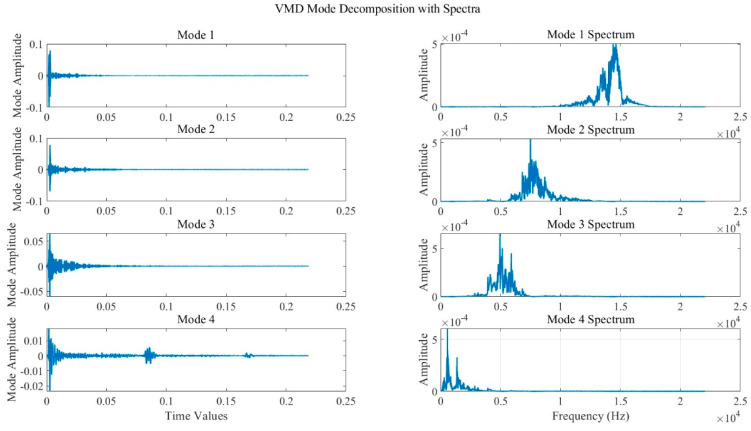
VMD of a normally empty pipe.

**Figure 6:**
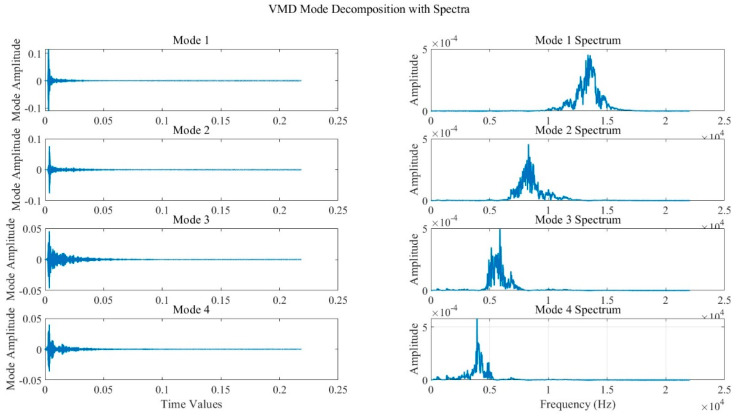
Decomposition of VMD with 20 mm blockage.

The VMD is performed for the normal empty pipe and the pipe containing 20 mm blockage, and the decomposition results are shown in Figure 5 and Figure 6. The two figures show that the center frequency of each IMF component has been completely decomposed without cross−mixing, and the decomposition method used above is reasonable.

**Figure 7:**
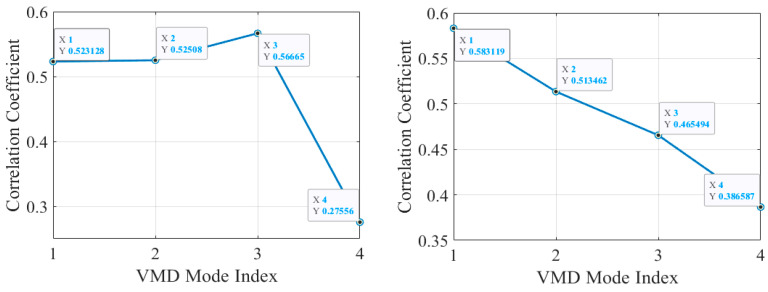
Plot of correlation coefficients for each component.

It is important to avoid pseudo−components in the analysis. The coefficients of the correlations between each component and the original signal were calculated to determine the effective modes after VMD. IMF components with correlation coefficients larger than half of the maximum correlation coefficient are selected, where the maximum correlation coefficient refers to the component that has the largest correlation coefficient within the original signal. Figure 7 shows the correlation coefficients for each IMF component. The graph displays the number of decompositions on the horizontal axis and the corresponding correlation coefficient values of each component on the vertical axis. All four IMF components of the decomposition have correlation coefficients larger than half of the maximum correlation coefficient, indicating that they should be retained.

In defect identification, the most representative information needs to be extracted from the data to describe the system state. Information entropy can be used to describe the complexity and uncertainty of data. In normal conditions, the system output has a certain degree of regularity, resulting in low information entropy. In other situations, however, the system output may become more random, leading to an increase in information entropy. Therefore, information entropy can be used as the main feature of the decomposed IMF components.

For the nine different operating states of pipelines collected in the experiments, the information entropy of each IMF component after VMD is extracted and the feature vector is constructed, and the results are shown in Table 4.

### 5.2. Model Implementation Details

#### 5.2.1. Attribute Matrix Construction

**Figure 8:**
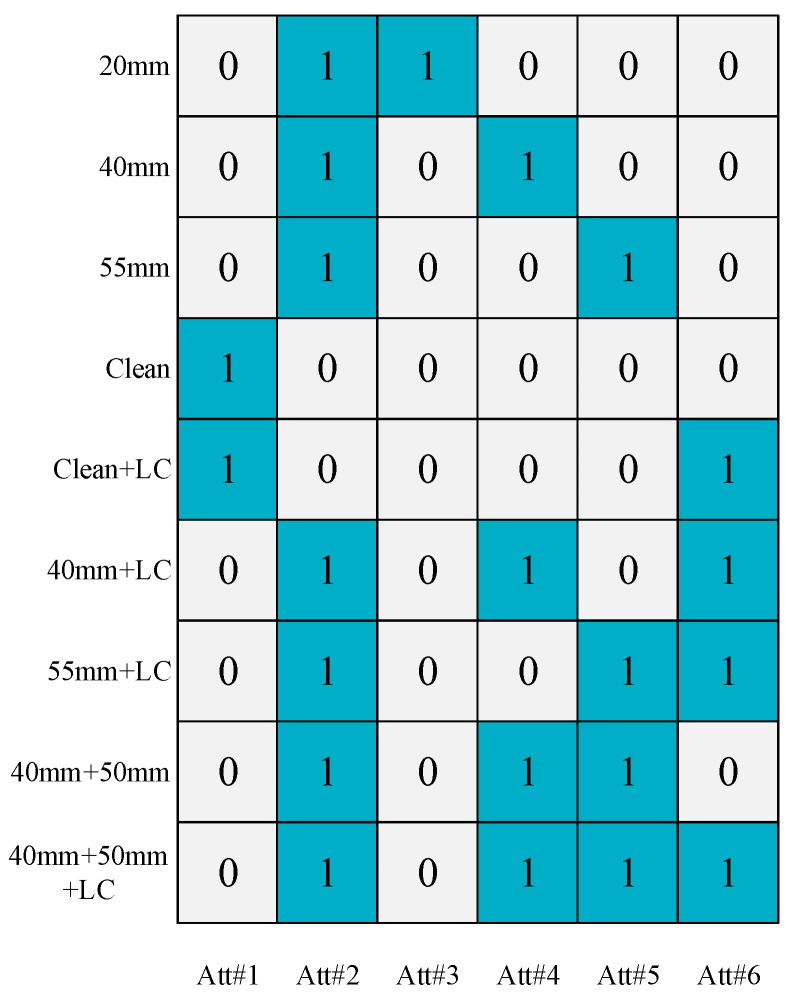
Attribute matrix of pipeline operational states.

Attributes are usually generated by manual definitions, which enable the differentiation of different defect classes by means of an accurate description of the defects. The occurrence of a class of defects may consist of one or more attributes that include the characteristics of the defect (e.g., the size and location of the defect) and the effects produced by the defect. In this paper, six attributes are defined in terms of the size of the blockage, the rate of fluid flow, and the presence or absence of a tee in the pipe (as shown in Table 5). Each defect and its six corresponding fine-grained attributes constitute the attribute matrix in zero-shot defect identification, in which “1” indicates that the defect has the attribute and “0” indicates that the defect does not have the attribute. Figure 8 shows the attribute matrix, with the attributes on the horizontal axis and the different operating states of the pipeline on the vertical axis. Take 20 of mm blockage as an example. When the blockage is 20 mm, the flow rate of the liquid in the pipe is slow and there is a light blockage, so attributes 2 and 3 are marked as “1”, while the other attributes are marked as “0”. This study uses manually defined attributes as auxiliary information to establish a link between known and unknown defect categories. By learning six fine-grained attributes, it is possible to train visible categories and classify and recognize unknown defect categories.

To ensure the reliability of the experimental process, the training and testing sets of the experimental data are randomly divided. Of the nine defect types, seven are randomly selected as training, and the remaining two are used as testing. The experimental data are randomly divided into six datasets (A, B, C, D, E, and F); each dataset has 350 (50 × 7) training samples and 100 (50 × 2) testing samples. In dividing the dataset according to the definition of zero-shot, it is guaranteed that the defect categories of the training set and the test set are completely different, and its specific division is shown in Table 6.

#### 5.2.2. Base Classifier Selection

**Figure 9:**
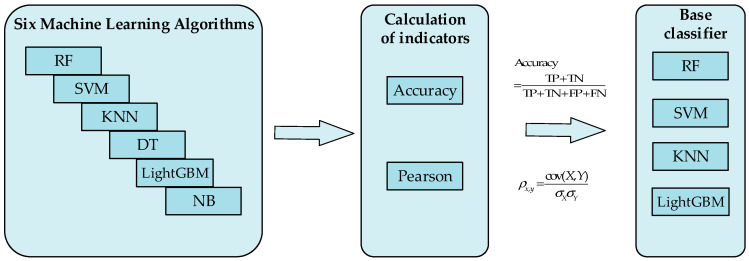
Flowchart of base classifier selection.

To construct the best attribute learner, complete the attribute learning and prediction of features, and obtain the best classification effect, it is necessary to make a reasonable selection of base classifiers for ensemble learning. To select suitable base classifiers, this paper conducts research in terms of both the accuracy and variability of classifiers. The chosen base classifiers must possess high classification accuracy, and there should be differences between the base classifiers. The base-classifier selection process is shown in Figure 9. Six machine learning algorithms, RF, SVM, KNN, DT, LightGBM, and NB, were selected to predict the classification accuracy of the model. Accuracy and the Pearson correlation coefficient were used as evaluation indicators. Based on the indicator results, four models with better effects were selected as base classifiers.

First, to ensure the fairness of the experiment, the key parameters of the base classifiers are optimized in this study using the grid search method. Using dataset A as an example, Table 7 shows the specific parameters of each model.

In Section 3.2.2 of this paper, RF, KNN, SVM, LightGBM, DT, and NB are initially selected as base classifiers. In this part, the authors performed an analysis and calculation of the classification accuracy of each model. Accuracy (Acc) is chosen to analyze the classification effect of the model, and its formula is as follows:

(9) $$Acc=\frac{TP+TN}{TP+TN+FP+FN}$$

where true positive (TP) represents the number of positives judged as positive, false negative (FN) is the number of positives misjudged as negative, false positive (FP) is the number of negatives misjudged as positive, and true negative (TN) is the number of negatives considered negative. The computational results of the classifiers at each level are shown in Table 8.

To improve the overall computational accuracy of the model, ensemble learning requires the base classifiers to have a certain level of accuracy. Table 8 shows that the NB model performs poorly in all six datasets, with an average recognition accuracy of only 0.497. The reason is that the NB model is based on the assumption of independence between features. This indicates that the model assumes that the features are independent of each other for a given category. In zero-shot learning, when there are unknown combinations of features, the model is unable to utilize the information from these combinations, resulting in poorer model classification. Therefore, the NB model with a lower accuracy has been removed.

In addition, there should be a certain difference between the selected base classifiers. Thus, the Pearson correlation coefficient is used to analyze the differences between models, and base classifiers with low correlation coefficients are preferred. The specific expression for calculating the Pearson correlation coefficient is as follows:

(10) $$\rho_{x,y}=\frac{\mathrm{cov}(X,Y)}{\sigma_{X}\sigma_{Y}}=\frac{E\left[\left(X-\mu_{X})(Y-\mu_{Y}\right)\right]}{\sigma_{X}\sigma_{Y}}$$

where cov denotes the covariance, X and Y denote two vectors, and $\mu_{X}$ and $\mu_{Y}$ are parameters.

**Figure 10:**
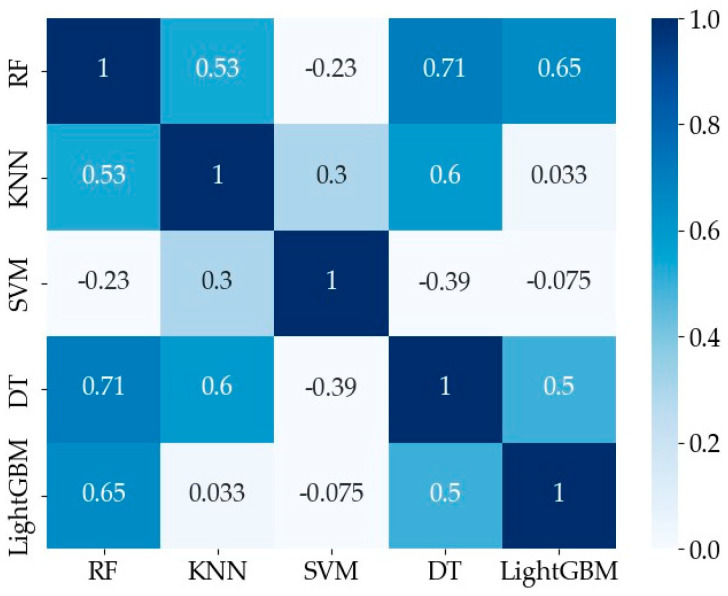
Model correlation analysis.

Figure 10 shows the correlation between the base classifier models. From the figure, we can see that the correlation between RF and DT is higher. Considering that the classification accuracy of DT is lower than that of RF, the DT model with a higher correlation and lower classification accuracy is removed, so the final selected base classifiers are RF, SVM, KNN, and LightGBM.

### 5.3. Comparative Analysis of Defect Identification Results

**Figure 11:**
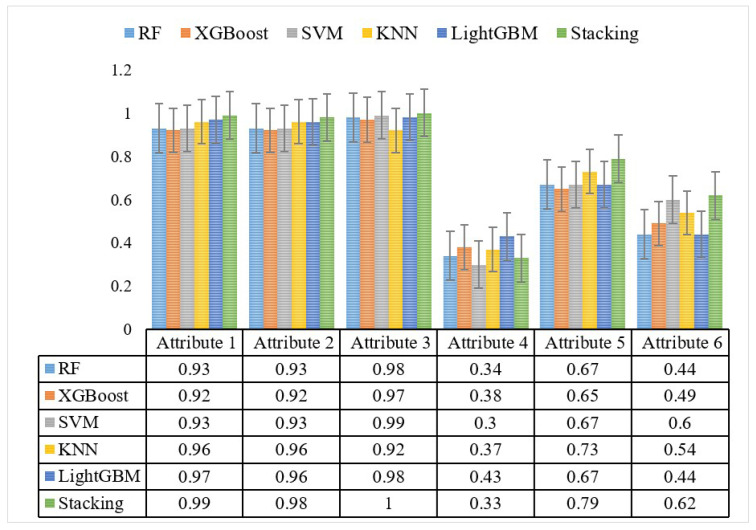
Attribute learning accuracy of models.

In defect identification, the model must first learn the attributes of the defect type, which is the basis of zero−shot learning. The accuracy of the model classification is directly affected by attribute learning. Therefore, this study evaluates the accuracy of attribute learning for defects using dataset A as an example. Figure 11 shows the attribute learning accuracy of different models.

Figure 11 shows that the different models have low learning accuracies for both attribute 4 and attribute 6 at around 0.5 or less. The reason for this is that attribute 4 (40 mm blockage) is very similar to attribute 3 (20 mm blockage) and attribute 5 (55 mm blockage) in terms of defect characteristics, therefore resulting in poor attribute learning. The feature difference is not obvious, which makes it difficult for the models to learn from the attributes. Meanwhile, the accuracy of attribute learning for attribute 6 (with the tee piece) is also low. This is because the tee is a pipeline branch connection, and when the branch diameter is small, the sound signal near the tee will not produce obvious energy accumulation like blockage, leading to more difficulties in identifying the tee.

**Figure 12:**
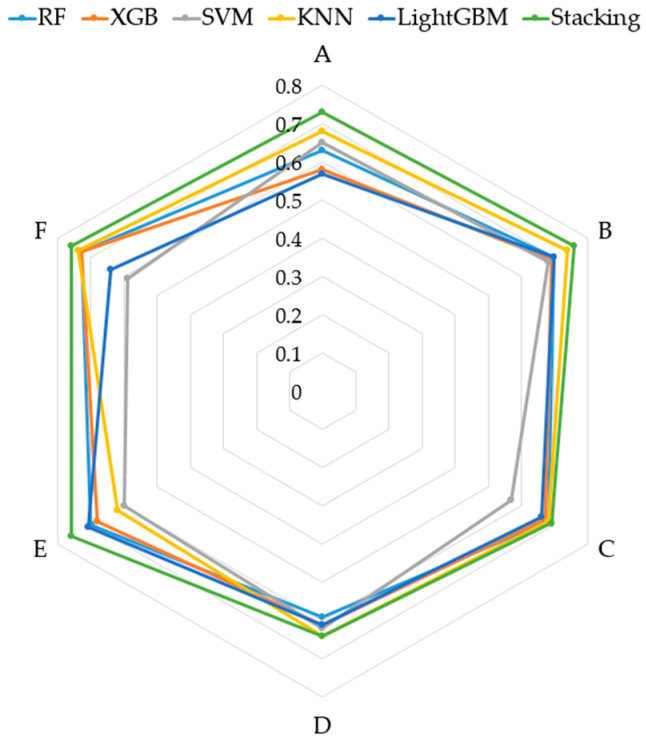
Comparison with single machine learning models.

To demonstrate the effectiveness of the proposed method, we conducted experiments on six datasets. The key parameter parameters of all models were optimized using a grid search algorithm, and the results of the comparison experiments are shown in Table 9, where the best classification results are marked in bold. Figure 12 is the radar chart of the accuracy results given in Table 9 and offers more intuitive visuals.

The above comparison experiments show that the proposed zero-shot defect identification ensemble model is better than the comparison model for classification on six datasets. The accuracy of the model on six datasets is 74%, 76%, 69%, 64%, 76%, and 76%, respectively, and the average accuracy is 72.5%, which is far superior to random guessing (50% accuracy). The experimental results demonstrate the feasibility of the zero-shot learning approach in pipeline blockage defect identification, which can be achieved by learning the attributes of the known defect categories to identify the unknown defect categories. Compared with the best-performing KNN model in the six datasets, the ensemble model improves the average accuracy by 4.5%; among the results, the best is achieved in dataset E, where the model accuracy is improved by 14%. In conclusion, for untargeted training samples and multiple types of underground pipe network defects, the method proposed is more robust and accurate than traditional machine learning models, and the method has higher application value and research significance for pipeline blockage defect identification.

**Figure 13:**
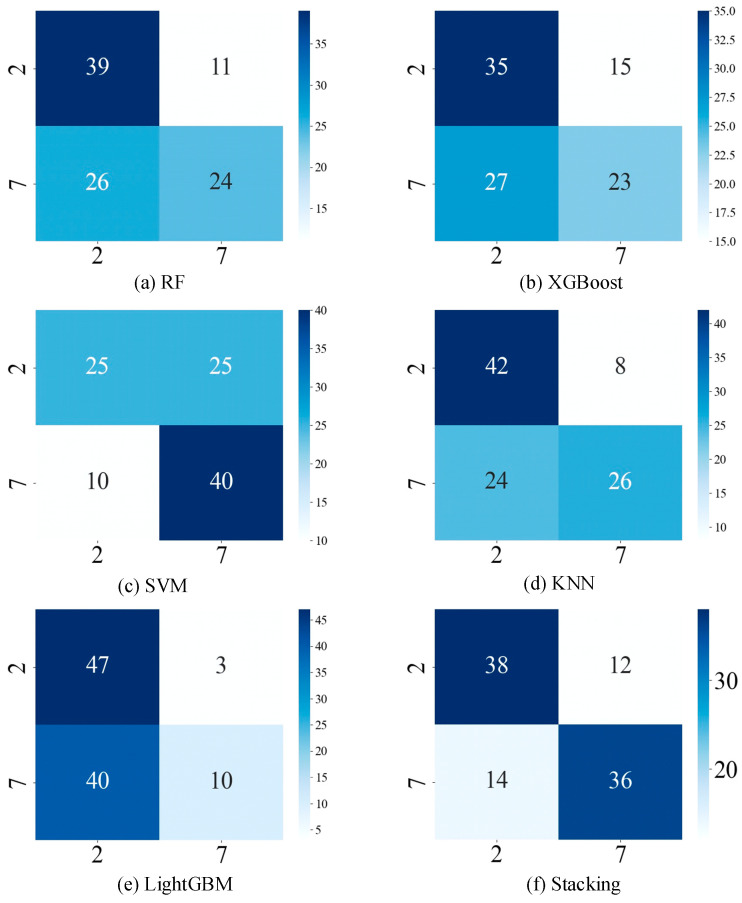
Confusion matrix of different models on dataset A. (**a**) RF. (**b**) XGBoost. (**c**) SVM. (**d**) KNN. (**e**) LightGBM. (**f**) Stacking.

Figure 13 shows the confusion matrix of each model on dataset A. The horizontal axis of the figure represents the predicted category of defects, the vertical axis represents the true category of defects, and the numbers represent the number of samples in which the defect category was classified as true or false. In the stacking ensemble learning model of Figure 13, 38 samples of defect 2 are correctly classified, and 12 samples are misclassified as defect 7. Similarly, 14 samples of defect 7 are misclassified as defect 2. There are a total of 100 samples of the two types of defects, of which 74 are correctly classified and 26 are misclassified. The confusion matrix shows that stacking ensemble learning is more effective in classifying the category of unknown defects in dataset A, with a 74% identification accuracy.

**Figure 14:**
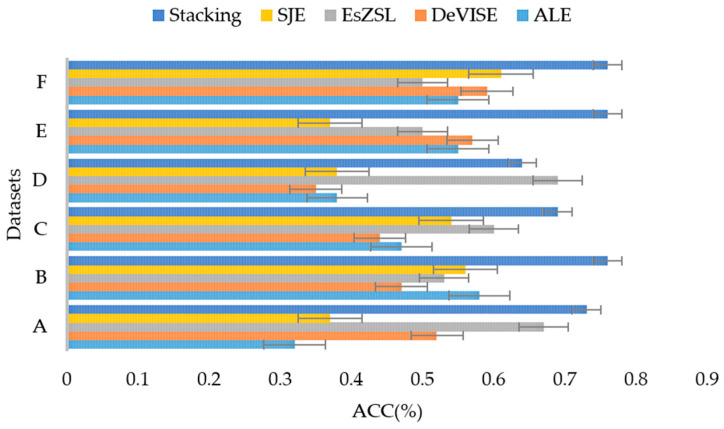
Comparison with zero-shot learning methods.

Meanwhile, to further illustrate the performance of stacking ensemble models, four classical zero-shot learning methods are compared: Attribute Label Embedding (ALE), Deep Visual-Semantic Embedding (DeVISE), Embarrassingly simple ZSL (EsZSL), and Structured Joint Embeddings (SJEs). The comparison models are described in Table 10. These four zero-shot learning methods are designed for the classification of two-dimensional image data, while the use of data is one-dimensional time series data, so the visual attributes in these four methods need to be replaced with manually defined defective attributes. The comparison results are shown in Table 11, with the best-performing model in each dataset marked in bold black font. Figure 14 is the bar chart of the accuracy results given in Table 11. In Figure 14, ACC on the horizontal axis represents the accuracy of model classification, and ABCDEF on the vertical axis represents the randomly divided data set.

From the above comparative experiments, we can see that for most groups, our method outperforms the other four ZSL methods. Since this paper randomly divides the data and the divided data groups are limited (6 groups), this may lead to limited feature expression in some datasets and insufficient feature learning in the model, resulting in accidental effects on the experimental results. Therefore, the proposed model was slightly weaker than the comparison model in individual groups in the comparison experiment, except for group D. The EsZSL model takes the importance of attributes into consideration and has better classification results than the other three zero-shot learning methods. For group D, the accuracy of the stacking ensemble model is slightly lower than that of EsZSL (by 5%), but for other groups, our method performs much better than EsZSL: 7% higher for group A, 23% higher for group B, 9% higher for group C, 26% higher for group E, and 26% higher for group F. Therefore, in the future, we can consider several aspects to minimize errors: 1. Add the attributes of defects to make the description of experimental objects more accurate; 2. Divide the experiment multiple times to reduce the impact of randomness. In conclusion, the traditional zero-shot classification methods based on visual attributes have rather poor performance on one-dimensional time series data, and the proposed stacking ensemble method is more applicable in this scenario.

## 6. Conclusions

The internal operating environment of buried drainage pipes is complex. Blockage, considered one of the main functional defects of the pipeline, is a gradually developing process. These characteristics of blockage development may result in a deficiency in the number of new defect types that appear between data collection cycles. As a result, these newly developed defects may lack sufficient labeled samples to train the identification model. A zero-shot pipeline defect identification method based on stacking ensemble learning is proposed. The identification task was accomplished by extracting effective features and learning attributes from the training data that did not contain the categories necessary for identification. The main conclusions are as follows:

1. We define attributes as accessorial information to establish links between different defect categories. The defect labels are transformed into an attribute matrix that applies to both the testing and training sets. Learning from the attributes of different categories, unknown pipeline defects can be identified, while the defect types are not included in the training dataset.
2. This study addresses the issues of low classification accuracy and poor robustness of traditional machine learning models facing a zero-shot learning task. This paper presents a stacking ensemble model that improves the accuracy of attribute learning by selecting base classifiers with higher accuracy rates and distinct differences between each other. The model showed better classification performance compared with other commonly used single-learning models on all six datasets.
3. Compared with the stacking ensemble learning model with traditional zero-shot learning models such as ALE and DeVISE, the comparison results indicated that traditional zero-shot learning is more effective on two-dimensional image data. However, the one-dimensional time series signals collected by industrial sensors do not contain visual attributes for the models to learn from. The method proposed is therefore more suitable for industrial defect identification applications.

In future work, we will explore advanced feature characterization methods to improve the accuracy of attribute learning and the classification performance for unknown defect types. Intelligent algorithms can be brought into the ensemble learning process to optimize the weight of base classifiers to make the ensemble model more effective. For many engineering applications, it is necessary to identify both known and unknown defects at the same time, and the idea of generalized zero-shot can be introduced in future research.

## Figures and Tables

**Table 1:** Description of pipeline operational status.

NO.	Pipeline Operating Status	Abbreviation
1	Pipeline with 20 mm blockage	20 mm
2	Pipeline with 40 mm blockage	40 mm
3	Pipeline with 55 mm blockage	55 mm
4	Normal empty pipeline	Clean
5	Normal empty pipeline with tee	Clean + LC
6	Pipeline with a tee and 40 mm blockage	40 mm + LC
7	Pipeline with a tee and 55 mm blockage	55 mm + LC
8	Pipeline with both 40 mm and 55 mm blockages	40 mm + 55 mm
9	Pipeline with tee and with 40 mm and 55 mm blockage	40 mm + 55 mm + LC

**Table 2:** Center frequencies of each IMF component for normal empty pipes.

K	Center Frequency/Hz				
	$IMF_{1}$	$IMF_{2}$	$IMF_{3}$	$IMF_{4}$	$IMF_{5}$
2	13,442.2	5863.53	\	\	\
3	13,442.2	8676.38	4939.64	\	\
4	13,442.2	8676.38	4929.64	**557.996**	\
5	13,899.6	114,480.1	7487.21	4939.64	**557.996**

**Table 3:** Center frequencies of each IMF component containing a 20 mm blockage.

K	Center Frequency/Hz				
	$IMF_{1}$	$IMF_{2}$	$IMF_{3}$	$IMF_{4}$	$IMF_{5}$
2	13,373.6	5881.83	\	\	\
3	13,373.6	8315.06	4935.07	\	\
4	13,373.6	8315.06	5881.83	**3928.84**	\
5	13,556.6	11,306.3	8315.06	5881.83	**3928.84**

**Table 4:** Information entropy of IMF components.

Defect Type	Information Entropy			
	$IMF_{1}$	$IMF_{2}$	$IMF_{3}$	$IMF_{4}$
20 mm	0.449	0.8415	0.9186	1.1217
40 mm	0.6576	0.9413	0.9277	1.3696
55 mm	0.8065	1.1675	1.1587	1.4284
Clean	0.7088	1.0124	0.9854	1.7658
Clean + LC	0.4757	0.5914	0.6992	1.3240
40 mm + LC	0.4014	0.6757	0.7331	1.6236
55 mm + LC	0.3456	1.2145	0.8336	0.9339
40 mm + 55 mm	0.4715	0.4586	0.7900	0.9446
40 mm + 55 mm + LC	0.4737	1.1921	0.7995	0.9410

**Table 5:** Attribute descriptions in the defect attribute space.

NO.	Attributes
Att#1	Flow rate normal
Att#2	Flow rate slow
Att#3	Mildly blockage
Att#4	Moderate blockage
Att#5	Serious blockage
Att#6	Pipe tee

**Table 6:** Training/test set division.

Dataset	Training Sets	Test Sets
A	1, 3, 4, 5, 6, 8, 9	2, 7
B	1, 2, 4, 5, 7, 8, 9	3, 6
C	1, 2, 3, 4, 6, 7, 8	5, 9
D	1, 3, 5, 6, 7, 8, 9	2, 4
E	1, 2, 3, 4, 5, 8, 9	6, 7
F	2, 3, 4, 5, 6, 7, 9	1, 8

**Table 7:** Set key parameters of a classifier.

Classifier	Parameter Value Settings
RF	max_depth = 6, min_samples_leaf = 5, min_samples_split = 10, n_estimators = 50
KNN	n_neighbors = 5, p = 1
SVM	kernel = rbf, C = 0.025
LightGBM	n_estimators = 250, learning_rate = 0.001, max_depth = 6
DT	max_depth = 25, min_samples_leaf = 2, min_samples_split = 5
NB	Default settings

**Table 8:** Classification accuracy of models on different datasets.

Classifier	Accuracy						Mean
	A	B	C	D	E	F	
RF	0.63	0.7	0.68	0.59	0.7	0.73	0.67
KNN	0.68	0.74	0.68	0.64	0.62	0.74	0.68
SVM	0.65	0.68	0.57	0.62	0.6	0.59	0.62
LightGBM	0.57	0.7	0.66	0.61	0.71	0.64	0.65
DT	0.49	0.66	0.68	0.58	0.58	0.72	0.618
NB	0.63	0.51	0.5	0.63	0.27	0.44	0.497

**Table 9:** Comparison with single machine learning models.

Methods	Accuracy						Mean
	A	B	C	D	E	F	
RF	0.63	0.7	0.68	0.59	0.7	0.73	0.67
XGBoost	0.58	0.69	0.67	0.61	0.68	0.73	0.66
SVM	0.65	0.68	0.57	0.62	0.6	0.59	0.62
KNN	0.68	0.74	0.68	**0.64**	0.62	0.74	0.68
LightGBM	0.57	0.7	0.66	0.61	0.71	0.64	0.65
Stacking	**0.74**	**0.76**	**0.69**	**0.64**	**0.76**	**0.76**	**0.725**

**Table 10:** Comparison model description.

Method	Description
ALE [35]	Considering image classification of attributes as a label embedding problem, a compatibility function is embedded between images and labels
DeVISE [36]	Semantic relations for sample labels are learned with textual data and images are explicitly mapped to a rich semantic embedding space
EsZSL [37]	The relationship between sample features, attributes, and classes is modeled as a two-layer linear network, and attributes are learned implicitly, the importance of attributes is considered, and finally, the recognition is performed using inner product similarity
SJE [38]	Constructing compatibility functions with multimodal semantic learning and different information embedding to improve classification accuracy

**Table 11:** Comparison with zero-shot learning methods.

Methods	Accuracy						Mean
	A	B	C	D	E	F	
ALE	0.32	0.58	0.47	0.38	0.55	0.55	0.475
DeVISE	0.52	0.47	0.44	0.35	0.57	0.59	0.49
EsZSL	0.67	0.53	0.6	**0.69**	0.5	0.5	0.582
SJE	0.37	0.56	0.54	0.38	0.37	0.61	0.472
Stacking	**0.74**	**0.76**	**0.69**	0.64	**0.76**	**0.76**	**0.725**

## Data Availability

Due to the nature of this research, participants of this study did not agree for their data to be shared publicly, and therefore these data are only available upon reasonable request.
